# Similarity from Multi-Dimensional Scaling: Solving the Accuracy and Diversity Dilemma in Information Filtering

**DOI:** 10.1371/journal.pone.0111005

**Published:** 2014-10-24

**Authors:** Wei Zeng, An Zeng, Hao Liu, Ming-Sheng Shang, Yi-Cheng Zhang

**Affiliations:** 1 Web Sciences Center, School of Computer Science and Engineering, University of Electronic Science and Technology of China, Chengdu, China; 2 State Key Laboratory of Networking and Switching Technology, Beijing, P.R. China; 3 Department of Physics, University of Fribourg, Fribourg, Switzerland; 4 Institute of Information Economy, Hangzhou Normal University, Hangzhou, China; 5 School of Systems Science, Beijing Normal University, Beijing, P.R. China; Université de Nantes, France

## Abstract

Recommender systems are designed to assist individual users to navigate through the rapidly growing amount of information. One of the most successful recommendation techniques is the collaborative filtering, which has been extensively investigated and has already found wide applications in e-commerce. One of challenges in this algorithm is how to accurately quantify the similarities of user pairs and item pairs. In this paper, we employ the multidimensional scaling (MDS) method to measure the similarities between nodes in user-item bipartite networks. The MDS method can extract the essential similarity information from the networks by smoothing out noise, which provides a graphical display of the structure of the networks. With the similarity measured from MDS, we find that the item-based collaborative filtering algorithm can outperform the diffusion-based recommendation algorithms. Moreover, we show that this method tends to recommend unpopular items and increase the global diversification of the networks in long term.

## Introduction

Nowadays, individuals are confronted with a large amount of contents such that it is very time-consuming to find the needed information, which is known as the information overload problem. This problem becomes more serious as the rapid development of the Internet. To solve this problem, many information filtering techniques, such as search engines and recommender systems, are widely investigated. Specifically, recommender systems are a newly emergent technique which predicts what a user likes based on his/her historical choices.

Up to now, many recommendation algorithms have been proposed such as collaborative filtering (CF) [1]–[3], matrix factorization [4], [5], spectral analysis [6], and so on. Some physical processes, including mass diffusion [7], [8], heat conduction [9], were also introduced by physicists to design recommendation algorithms. A detailed summarization of recommender system technologies can be found in [10]. The most significant finding from these diffusion-based methods is that the hybridization of the mass diffusion and heat conduction can achieve both accurate and diverse recommendation [11]. This pioneer work was followed up later with many extensions such as the semi-local diffusion [12], the preferential diffusion [13], the biased heat conduction [14], network manipulation [15] and the item-oriented method [16]. Recently, the long-term influence of these diffusion-based recommendation methods on network evolution has also been studied [17], [18].

Among the aforementioned algorithms, CF has been successfully applied in e-commerce [19], [20]. The CF actually have two different versions: the user-based CF (UCF) and the item-based CF (ICF) [21]–[24]. The user-based CF estimates each user's preferences by referring to her similar users' tastes, while the item-based CF recommends items which are similar to the target user's selected items. Generally, the accuracy of the item-based CF is higher than that of the user-based CF. For both algorithms, the most important issue is how to qualify the similarities between users or items. There are many methods to measure the similarities of nodes based on network structure analysis including common neighbors, cosine index, Katz index, just to name a few [25], [26]. However, these simple structural-based similarity measures are usually sensitive to the noisy information in networks, which results in a low recommendation accuracy. Moreover, some of these measures are strongly biased to large degree items, which makes the unpopular but relevant items be overlooked in the recommendation [3].

To solve the problems above, we make use of the multidimensional scaling (MDS) technique to estimate similarity between nodes. Online user-item bipartite networks are represented by a 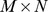 adjacency matrix where 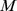 and 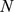 are respectively the number of users and items. Therefore, each item is described by a *M-dimensional* vector from the adjacency matrix. Based on MDS, we design a method to map the *M-dimensional* item vectors into *H-dimensional* item vectors (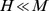) and compute the similarities of item pairs in the *H-dimensional* space. There are two advantages: (1) The noise of data can be diminished by the dimension reduction, so that the similarity based on the low-dimensional space is more accurate than the high-dimensional space [27], [28]. We compare the MDS method with the commonly-used cosine method in both artificial and real data, and find that the MDS method significantly outperforms the cosine method in estimating the item similarity. (2) MDS can remarkably speed up the computation of item similarity since we only have to deal with *H*-dimensional item vectors. Therefore, the MDS method can be used in the large-scale dataset. In fact, some other dimension reduction methods such as matrix factorization (MF) and singular value decomposition (SVD) have also been used in recommender systems [29], [30]. In both methods, not only the item vectors but also the user vectors are considered. In most online systems such as user-movie rental systems, the number of users significantly exceeds the number of items. Therefore, it requires much more memory to store the user vectors than the item vectors. In other words, the MDS requires much less memory than the MF and SVD, making it more scalable.

We further apply the MDS to the item-based collaborative filtering algorithm. We test this method on real datasets and the results show that our method enjoys a considerably higher recommendation accuracy and diversity than the diffusion-based recommendation methods. Moreover, by investigating the network evolution driven by the recommendation algorithms, we found that our method could result in a more homogeneous item degree distribution in the long term.

## Methods

### Collaborate Filtering

A recommender system can be naturally described by a user-item bipartite network with the adjacency matrix 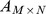 in which the element 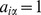 if the user 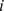 has collected the item 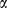, and 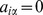 otherwise (To be consistent with previous papers, we use Greek and Latin letters, respectively, for item- and user-related indices) [2], [31]. 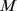 and 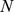 are the number of users and items, respectively. The performance of ICF and UCF depends a lot on the similarity definition and the data sets [24], [32]. We mainly focus on ICF in this paper, but parallel techniques can be applied in a user-oriented fashion.

The ICF provides each individual user with items which are similar to her selected items. That is, for user *i*, the recommendation score of item 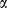 is
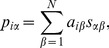
(1)where 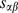 is the similarity between item 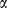 and *β*. Items will be sorted in descending order according to 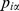 and the top-*L* items will be recommended to *i*. The most common way to compute 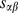 is the cosine index [33], [34], that is,
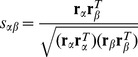
(2)where 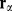 and 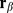 are the 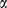 and 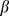 column of adjacency matrix *A*, respectively. This combination is referred as the ICF-cosine. There are some drawbacks of the standard cosine index and the ICF based on this index have some potential risks, which we will discuss later in the section.

### Multi-dimensional Scaling

In bipartite networks, each item is characterized by the corresponding column of the adjacency matrix *A*, i.e. a *M*-dimensional vector. The goal of the MDS is to map the *M-dimension* vectors 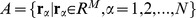 into the *H-dimension* vectors 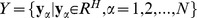, such that dissimilarities from *M-dimension* space 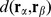 are well-approximated by the distances in the lower *H-dimensional* space 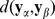. The input of the MDS is an item × item dissimilarity (or similarity) matrix 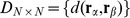. One simple way to compute the 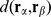 is the Euclidean distance: 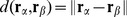. Given the dissimilarity matrix *D*, the task of the MDS is to minimize the cost function
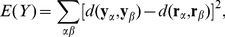
(3)where the 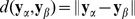 is the distance of item 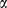 and 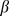 from the *H-dimension* space. A well-known approach to find the solution is the Gradient Descent (GD) algorithm which repeatedly processes the iteration:



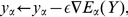
(4)Where
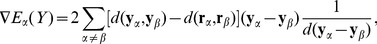
(5)and 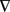 is the gradient operator. The step size 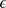 should be small enough (e.g. 0.005).

Another kind of MDS takes into account the rank-order of the dissimilarities. That is, the Euclidean distances between points in *Y* approximate a monotonic transformation of the corresponding dissimilarities in *D*. Therefore, the cost function of this method is
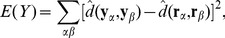
(6)where 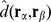 is the monotonic transformation of 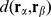 using a least squares monotone regression algorithm called monotone fitting (MFIT), which is described in ref [35]. The MDS based on equation 3 is called *Metric MDS* (MMDS for short) and that based on equation 6 is called *Non-Metric MDS* (NMDS for short).

When recommending items to users, we apply the MDS (MMDS and NMDS) to measure the similarities of item pairs and then compute the recommendation score between user 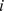 and item 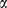 by equation 1. We refer this method as ICF-MDS. All *i*'s uncollected items are sorted in descending order according to 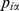 and the *top-L* items will be recommended to user *i*.

### Diffusion-based Methods

The diffusion-based recommendation algorithms are commonly considered as the state-of-the-art approaches in both accuracy and diversity. The most representative one is the hybrid method (short for Hybrid) [11] which combines the mass diffusion (short for MD) [7] and heat conduction (short for HC) [9] processes. The hybrid method starts by assigning 1 unit resource to each selected item of the target user, and 0 to the unselected items. Denoting the initial resource vector as 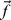, the resources will then diffuse in the user-item bipartite network according to 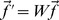 where 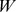 is the diffusion matrix with each element
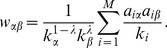
(7)


In above equation, 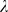 is a tunable parameter. If 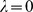, it degenerates to the pure HC algorithm [9]. If 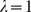, it gives the MD algorithm [7]. The final resource vector 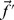 will be sorted in the descending order and those items with most resources will be recommended.

In fact, the hybrid method is the same type of method as the cosine method. Given two items 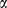 and *β*, their cosine similarity is 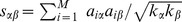. For the diffusion-based recommendation methods, the diffusion of resource on bipartite networks actually aims to calculate the similarity between items. Take the hybrid method as example, the resource that 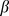 receives from 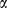 reads 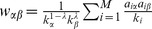, where 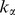 and 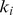 are the degree of item 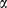 and user *i*, respectively. 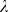 is a tunable parameter. 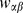 can be considered as the “similarity" between 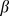 and *α*. The cosine method is based on the calculation of the scalar product between two vectors. So the hybrid method can be regarded as a weighted scalar product between two vectors. Though there is an obvious commonness between these two methods, there is one important difference between them: the 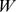 matrix in the hybrid method is asymmetric while the 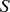 in the cosine method is symmetric. Different from the hybrid and cosine method, the MDS is based on Euclidean distance between two vectors. In principle, the distance between two vectors can be defined in other ways. We thus tried other distance definition in MDS, such as Euclidean Commute-Time Distance [36] and Hamming distance. We found that the Euclidean distance works best among these three (See table S3 in File S1).

### Metric

The MovieLens data is used to test the algorithms' accuracy and diversity, which consists of 6040 users, 3900 movies and 1 million links (See table S1 in File S1). The results on other datasets are consistent with Movielens and presented in the supporting information material (See Fig. S1, Fig. S2 and table S4 in File S1). The data is randomly divided into two parts: the training set (*E^T^*) and the probe set (*E^P^*). The training set contains 80% of the original data and the recommendation algorithm runs on it. The rest of the data forms the probe set, which will be used to examine the recommendation performance. Measuring the accuracy and the diversity of top-*L* items in individuals recommendation list is actually more important from practical point of view since in real recommender systems individuals are only presented with top-*L* items. Accordingly, we employ four different metrics to measure accuracy and diversity of the top-*L* recommendation. A brief description of these four metrics is shown as follows:

#### Precision

For a target user *i*, the precision of recommendation, 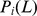 is defined as 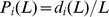, where the 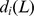 is the number of hit links, namely user *i*'s associated links that are contained by both the probe set and the top-*L* recommendations. The precision of the whole system is the average of individual precisions over all users, given as 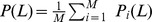.

#### Recall

The recall of recommendation to *i*, 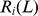, is defined as 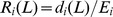, where 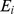 denotes the number of 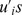 links in the probe set. Similarly, the recall of the whole system is defined as 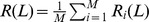. Higher precision and recall indicate higher accuracy of recommendations.

#### Hamming distance

This metric considers the uniqueness of different users' recommendation list. Given two users 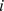 and *j*, the difference between their recommendation lists can be 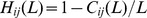, where 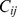 is the number of common items in the *top-L* places of both lists. Clearly, if user 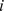 and 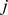 have the same list, 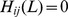, while if their lists are completely different, 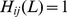. Averaging 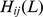 over all user pairs we obtain the mean distance 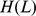, for which greater or lesser values mean, respectively, greater or lesser personalization of users' recommendation lists.

#### Novelty

This metric concerns the capacity of recommender systems to generate novel and unexpected results. Given an item *α*, its novelty is 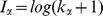. From this we can calculate the mean novelty 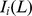 of each user's *top-L* items, and averaging over all users we obtain the mean novelty of the system 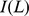.

## Results

In this section, we will discuss the performance of MDS in estimating item similarities in both the artificial data and real data. For item 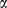 and *β*, one can get their similarity from the *M-dimension* space by equation 2. Their similarity from the *H-dimension* space can be obtained based on the 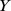 computed by the MDS. That is,
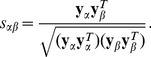
(8)


By comparing these two methods, one can identify which one performs better in quantifying the similarities between items. We normalized the similarities as follows:
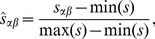
(9)where 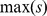 and 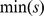 are the maximum and minimum of all the similarities, respectively.

### Simulations in Real Data

**Figure 1:**
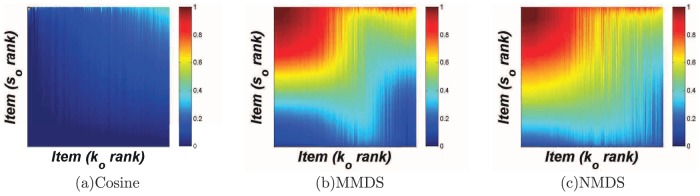
The compare of *cosine* and *MDS* (MMDS and NMDS) method in real data, MovieLens. All the movies are sorted by their degrees in a ascending order (horizontal ordinate). For a given movie 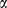, other movies are sorted by their similarities with 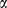 in a ascending order (vertical ordinate) and the color depth denotes the value of similarity.

We carried out the simulations in an artificial data which consists of 500 users and 500 items. The results show that both MMDS and NMDS are significantly more accurate than the cosine method in estimating similarity between items (See Fig. S1 in File S1). We further compare the cosine and the MDS method on a real online bipartite network called MovieLens. The original data consists of 6040 users, 3900 movies and 1 million ratings. The rating matrix is transformed to *0–1* matrix where 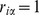 if 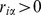. We randomly select 500 movies and compute their similarities by the cosine, MMDS and NMDS methods, respectively. All the similarities are normalized by equation 9 and reported in Fig. 1. The movies are sorted according to their degrees in the ascending order. That's to say, the movies' degree increases from the left to the right in Fig. 1. For each movie, we then sort its similarities with other movies in the descending order, i.e., the value of similarity decreases from the top to the bottom in Fig. 1. The color denotes the value of similarity.

One can see from the Fig. 1 that most similarities from the cosine method range from 0 to 0.2 and only a few of them are larger than 0.5, which indicates that the similarities between items are not well distinguished. The obtained item similarities based on the MMDS and NMDS share the same properties: Firstly, for each movie, its similarities with other movies vary significantly. Secondly, for those unpopular movies, their similarities with other movies tend to be very high and some of them are close to 1. But for those popular movies, their similarities with other movies are smaller. One possible reason is that the large degree movies have been collected by many users with different preferences. As a result, it is very difficult to identify which categories those movies belong to. Accordingly, their similarities with other movies are small.

**Figure 2:**
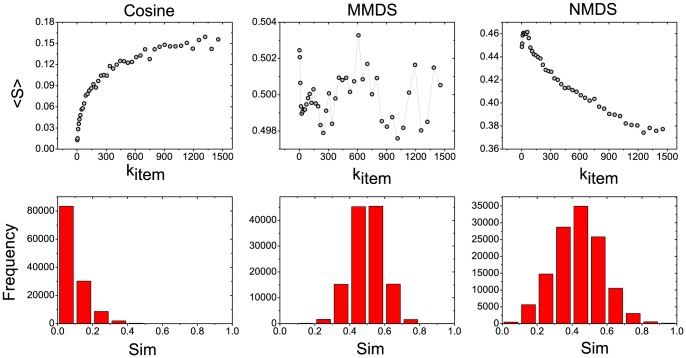
The relationship between average similarity of an item to other items and its degree, as well as the distributions of similarity scores under different methods.

Moreover, we present the relationship between average similarity 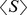 of an item to other items and its degree 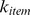 in the top three figures in Fig. 2. It can be seen that in the cosine method 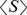 increases with 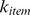. In the MMDS method, 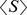 is roughly independent of 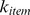. In NMDS, 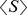 decreases with 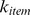. The distribution of similarity scores is also presented in the bottom three figures in Fig. 2. One can see that the similarity scores in the three methods are all homogeneously distributed. The mean of the distribution is around 0.5 in MMDS and NMDS, while the mean of the distribution is much smaller (around 0.1) in the cosine method.

### Recommendation Accuracy and Diversity

We study the relationship between the accuracy and the dimension of 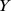 computed by the MDS (See Fig. S2 in File S1). Our results show that the accuracy cannot be constantly increases by increasing 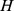 (when 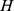 is large enough, further enlarging 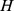 only includes noisy information). We also compared MDS to a matrix factorization method called the singular value decomposition (SVD) [37]. The SVD uses the 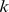-largest singular values of 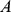 to construct a matrix 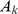 to approximate 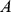. Here 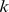 is also the dimension of the obtained vectors from the decomposition. Normally, the optimal parameter 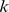 is determined by the number of largest singular values that are significantly larger than 0 [37]. After applying SVD to the movielens data, the results show that the singular value is close to zero when the dimension 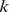 exceeds 50. However, the best dimension number of the MDS method is around 100 (See Fig. S2 in File S1). The best dimension number obtained from SVD is different from that from MDS. This may due to the fact that the best dimension number in SVD and MDS (with ICF) is determined by different mechanisms: 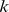 in SVD is determined by the largest-singular values while 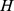 in MDS is determined by the recommendation precision.

We further compare our methods with the diffusion-based recommendation algorithms and the results are presented in the table 1. The accuracy of HC method is the lowest among these methods since it overwhelmingly focuses on the diversity of recommendation. The ICF-cosine is better than the MD but it is less effective than the Hybrid method. Among all the considered recommendation methods, the ICF-MMDS achieves the highest accuracy. More specifically, the ICF-MMDS method outperforms the ICF-cosine method by 19.7% and 27.9% in *precision(L = 10)* and *recall(L = 10)*, respectively. These results confirm our previous conclusion that the similarity based on the MDS is better than the cosine index. We also carried out the simulation to compare MDS and cosine similarities under the UCF framework. Our experimental results show that UCF-MDS has higher recommendation accuracy than UCF. However, UCF-MDS is less effective than ICF-MDS (See table S2 in File S1).

**Table 1:** The accuracy compare results of different recommendation approaches on MovieLens.

Method	Precision(*L* = 10)	Precision(*L* = 20)	Recall(*L* = 10)	Recall(*L* = 20)
ICF-MMDS	**0.3507**	**0.2844**	**0.1604**	**0.2412**
ICF-NMDS	0.3338	0.2716	0.1506	0.2284
MD	0.2355	0.1900	0.1006	0.1528
HC	0.0024	0.0235	0.0014	0.0186
Hybrid	0.3256	0.2673	0.1492	0.2325
ICF-cosine	0.2929	0.2323	0.1254	0.1853

The recommendation length *L* is set to 10 and 20. The dimensions of both ICF-MMDS and ICF-NMDS are 100. The 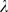 of Hybrid method is 0.2.

**Figure 3:**
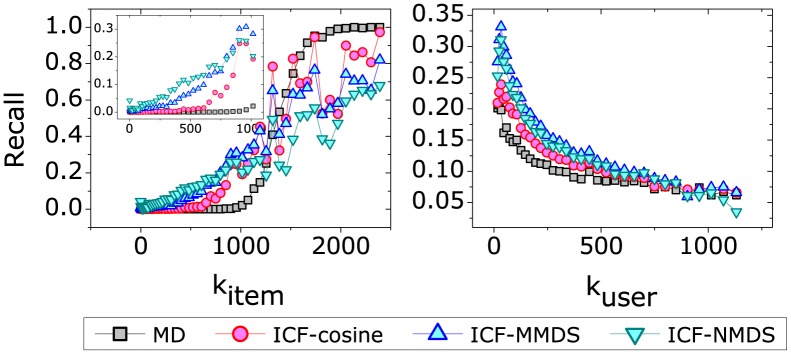
The relationship between accuracy and the user degree (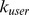) and movie degree (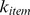). For a given *x*, its corresponding 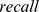 is obtained by averaging all the users whose degrees are in the range of 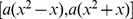, where 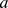 is chosen as 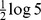. The recommendation length is 20 and the dimension of MMDS and NMDS is set to 30.

In order to give more details about the ICF-MMDS and ICF-NMDS method, we study in detail the recommendation accuracy on users and items with different degrees. Since recall is defined based on users, it can be naturally used to measure the recommendation accuracy of the users with the same degree. When applied to items, we define the item recall as: 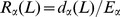 where 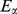 is the number of users who selected item 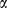 in the probe set, and 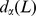 is the number of times that 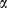 appears in these 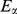 users' recommendation lists. The recall of the items with the same degree is obtained by simply averaging 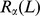 of these items. The left figure of Fig. 3 gives the relationship between the accuracy and the movie degree. As one can seen, both ICF-MMDS and ICF-NMDS significantly improve the accuracy of small degree movies. Among all the methods, MD performs worst in recommending small degree movies. The right figure of Fig. 3 shows the relationship between the accuracy and the user degree. It can be seen that the ICF-MMDS and ICF-NMDS methods outperform others for both small and large degree users.

**Figure 4:**
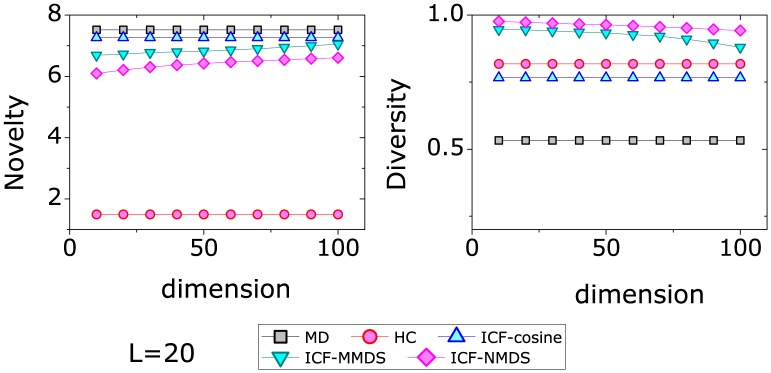
The diversity results of different recommendation approaches on MovieLens. The recommendation length is set to 20.

Our above results show that the ICF-MMDS and ICF-NMDS can improve the accuracy of those unpopular movies, which implies the recommendation from these two methods are diverse. The novelty and diversity results of those methods on MovieLens are presented in Fig. 4. The left figure gives the results of *Novelty*, where it can be seen that the best method with respect to *Novelty* is HC. On the contrary, the *Novelty* of the MD and ICF-cosine are not satisfactory enough. The *Novelty* of ICF-MMDS and ICF-NMDS increases with the dimension *H*, which indicates that they provide more novel movies with a smaller *H*. The right figure gives the recommendation diversity measured by the *Hamming Distance*. Different from the *Novelty*, the best method is the ICF-NMDS rather than the HC method. The diversity of both ICF-MMDS and ICF-NMDS methods decreases with the dimension *H* but still better than others when *H* is large.

### Effect on Network Evolution

**Figure 5:**
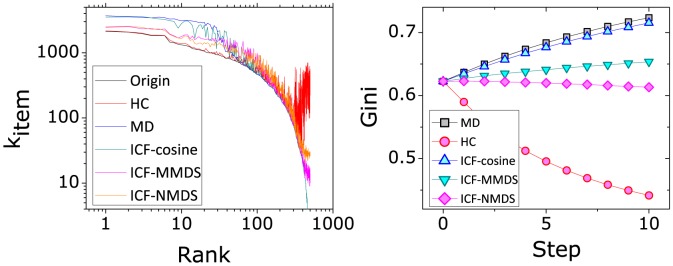
The changes of each movie's degree and the *Gini* index of the system. The dimension of MMDS and NMDS is set to 30.

Moreover, we study impacts of recommendation algorithms on the long-term diversification of user-item bipartite network. We again randomly sample 500 movies from the MovieLens data. For each user, we provide her with top-10 ranked movies by the recommendation algorithm and assume that she will randomly select one of them. As a result, each user's degree will be increased by 1. We repeat this scenario for 10 times and then investigate the changes of each movie's degree distribution as well as the corresponding *Gini* coefficient. The left figure of Fig. 5 gives the changes of movies' degrees in the zipf plot. The *Origin* curve denotes movies' degrees in the original bipartite network. Other curves denote the movies' degrees after 10 times of the above recommendation processes. We observe that the top-100 popular movies' degrees are greatly increased by the MD and ICF-cosine algorithms while the degree increment of other movies is very small. It means the unpopular movies are overlooked while popular movies are mostly recommended by these two methods. The HC algorithm mainly increases the degrees of those unpopular movies, which is opposite to the algorithm of MD and ICF-cosine. Different from the previous methods, the degrees of both the popular and unpopular movies are increased by the ICF-MMDS and ICF-NMDS. Between ICF-MMDS and ICF-NMDS, one can see that the degree increment of unpopular movies by the ICF-NMDS is more than that by the ICF-MMDS, which indicates that the ICF-NMDS works better in recommending the fresh movies for users.

The changes of *Gini* coefficient of the system is presented in the right figure of Fig. 5. Suppose **k** is the movie degree vector sorted in the ascending order, the *Gini* coefficient of the system is
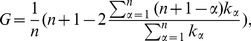
(10)where 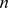 is the size of **k**. The *Step* in the figure denotes the number of iterations of the above recommendation process. If *Step*  = 0, the *Gini* coefficient is computed by the original movie degrees. For MD and ICF-cosine, the Gini coefficient grows fast after each recommendation process. This “rich gets richer” result in fact contradicts to the concept of personalized recommendation which is supposed to guide users' attention to different items according to their personal tastes. The HC algorithm decreases the 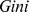 of the system in the long-term since it mainly recommends those unpopular movies to users. For both the ICF-MMDS and ICF-NMDS, the *Gini* coefficient stays relatively stable in long term.

### Complexity of Recommendation Algorithms

We finally discuss the computational complexity of our methods. The complexity of computing the distance matrix is 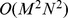 where 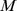 and 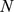 are the number of user and items, respectively. There are 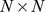 entries in the distance matrix, therefore the complexity of computing the low-dimension matrix 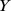 by the gradient descent method is 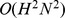 where 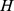 is the dimension of *Y*. We test the methods on an i5-2500 dual-core processor 3.3 GHz PC. For the MovieLens data set, it spends 571.6 s in total to compute the 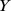 by the MDS method and only 0.6041 s to calculate the similarities over all item pairs when *H* = 100. However, it takes 340.9 s to compute item similarities by the traditional cosine method. From the definition of mass diffusion and hybrid method, they have the same computational complexity with CF method as the resource diffusion process can be considered as the computation of item similarities. To obtain the transition matrix, it takes 319.8 s and 525.2 s for the mass diffusion and hybrid method, respectively. Although the total running time of the MDS-based method is more than the traditional methods, the computation of 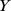 can be done off-line. When providing on-line recommendation service for users, we can use the pre-stored 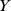 to calculate the item similarities and recommend items by CF method.

**Figure 6:**
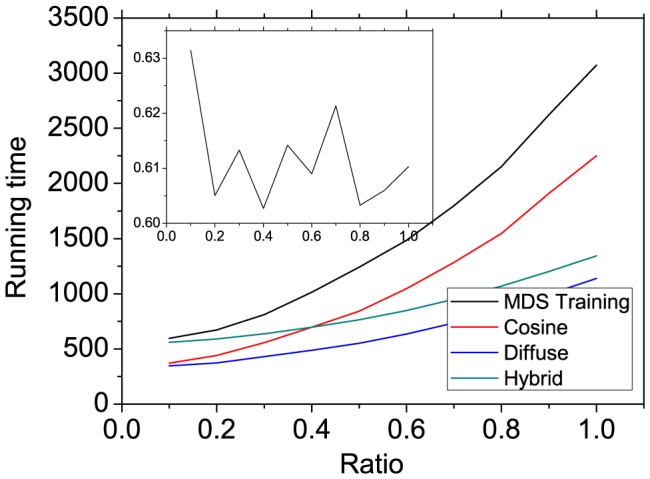
The computation time of methods with the increasing of network size. Inset gives the running time of computing the item similarity matrix by Y when the dimension number is 100.

Additionally, we show in Fig. 6 the computation time of different methods when the network size is increased. Starting from the real data, we add some ratio of artificial users with degree equal to the mean degree of the existing users. The links of new users randomly connect to the items. Fig. 6 shows the relation between the computation time for the item similarity and the ratio of new users. From the figure, one can see that the computation time of traditional methods (cosine, diffuse and hybrid) increases with the number of new users in the system. Although the running time of MDS training process (computing Y matrix) is increased with the user number, the running time of computing the item similarity matrix is barely affected, as shown in the inset in Figure 6. As we discussed above, the computation of Y matrix can be done off-line and the computing the item similarity matrix is done online. Therefore, the recommendation speed of the ICF-MDS method is independent of user number in real application.

## Discussion

The collaborative filtering method is considered as the most popular and already widely applied to e-commerce. The performance of CF strongly depends on the approach of computing the users' or items' similarity. In the literature, there are many handy similarity measures such as common neighbor index and its variants. However, theses methods cannot smooth out noise, which may result in a distorted estimation of the similarity between nodes. To solve this problem, we apply the multi-dimensional scaling method to measure similarity. The method first maps the items from high dimension to low dimension, then compute the item similarity from the low dimension space. This mapping process can effectively eliminate the noisy information from data and result in a more accurate recommendation when applied to item-based collaboration filtering method. Moreover, the computing complexity of similarity from the low-dimension space is much lower than that from the high-dimension space, which efficiently accelerates the speed of recommendation. Finally, we study the long term diversification of the resulted bipartite networks when different recommendation methods are repeatedly used. We find the ICF based on MDS can lead to a relatively stable degree distribution of the items, which may help to form a healthy information ecology in practice.

## Supporting Information

File S1
**Combined file of supporting figures and tables. Figure S1.** The compare of *cosine* and *MDS* (MMDS and NMDS) method in artificial data. The dimension 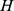 of 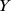 is increased from 2 to 50. **Figure S2.** The relationship between the accuracy (precision and recall) and the dimension 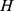 of 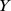 computed by the MDS. The recommendation length is 20. **Figure S3.** The relationship between the accuracy (precision and recall) and the dimension of 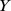 computed by the MDS on Netflix and RYM dataset. The recommendation length 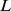 is 20. **Figure S4.** The diversity and novelty results on the Netflix and RYM data. The recommendation length 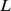 is 20. **Table S1.** The MDS-based method under the UCF framework. The dimensions of ICF-MDS and UCF-MDS are 100 and 200, respectively. **Table S2.** MDS based on different distance computations.(PDF)
