# Supplementary material for: Similarity from Multi-Dimensional Scaling: Solving the Accuracy and Diversity Dilemma in Information Filtering
Source: PLoS One. 2014 Oct 24;9(10):e111005. doi: 10.1371/journal.pone.0111005 (PMC4208813; doi:10.1371/journal.pone.0111005)
Supplement: File S1 — Combined file of supporting figures and tables. Figure S1. The compare of cosine and MDS (MMDS and NMDS) method in artificial data. The dimension of is increased from 2 to 50. Figure S2. The relationship between the accuracy (precision and recall) and the dimension of computed by the MDS. The recommendation length is 20. Figure S3. The relationship between the accuracy (precision and recall) and the dimension of computed by the MDS on Netflix and RYM dataset. The recommendation length is 20. Figure S4. The diversity and novelty results on the Netflix and RYM data. The recommendation length is 20. Table S1. The MDS-based method under the UCF framework. The dimensions of ICF-MDS and UCF-MDS are 100 and 200, respectively. Table S2. MDS based on different distance computations. (PDF) [file pone.0111005.s001.pdf]

Supplementary Information
Similarity from multi-dimensional scaling: solving the accuracy and diversity dilemma in information filtering

Wei Zeng, An Zeng, Hao Liu, Ming-Sheng Shang and Yi-Cheng Zhang

Data

We present here the recommendation results of different algorithms in three real networks, namely MovieLens, Netflix and RYM. MovieLens (http://www.grouplens.org) is a movie recommendation website, which provides personalized recommendations by using users' historical ratings. The data used in this paper consists of 6040 users and 3900 movies. Netflix (http://www.netix.com) is an online DVD and Blu-ray Disc rental service in the US. The data we used is a random sample that consists of 3000 users and 3000 movies. RYM (http://rateyourmusic.com/) is an online community of people who love music and allows individuals to catalog, rate, tag and review music. The data used in this paper is also a random sample consisting of 33762 users and 5267 songs. More details about these datasets are presented in table S1.

The data is randomly divided into two parts: the training set (E^T) and the probe set (E^P). The training set contains 80% of the original data and the recommendation algorithm runs on it. The rest of the data forms the probe set, which will be used to examine the recommendation performance. We apply *precision* and *recall* to measure the accuracy of recommendation algorithms (see definition in the main manuscript). Higher precision and recall indicate higher accuracy of recommendations. The *hamming distance* are chosen to measure the diversity of recommendation list and the *novelty* are selected to measure the capacity of the recommender system to generate novel and unexpected results (see definition in the main manuscript).

The relationship between the accuracy (precision and recall) and the dimension d of Y computed by the ICF-MDS on Netflix and RYM dataset is presented in figure S1. The accuracy of other recommendation approaches is presented in table S2. The diversity and novelty results on the Netflix and RYM are presented in figure S2.

Table S1. The statistics of datasets. The sparsity is obtained by $\frac{\#links}{M\times N}$, where N and M are the number of users and items, respcetively.

Dataset	#user	#items	#links	sparsity
MovieLens	6040	3900	1,000,000	4.3%
Netflix	3000	3000	197,248	2.2%
RYM	33762	5267	606,018	0.3%

Simulations in Artificial Data

Our artificial data consists of 500 users and 500 items. The distributions of the user degree (the number of items the user has collected) and the item degree (the number of users who have collected the item) follow the power-law form $k^{-2.5}$. To generate connections between users and items, we assume that each user i has a taste vector with *U-dimension*, $\mathbf{p}_i = (p_{i,1}, p_{i,2}, ..., p_{i,U})$ [1]. Similarity, attributes of the item α are represented by the *U-dimensional* attribute vector $\mathbf{q}_\alpha = (q_{\alpha,1}, q_{\alpha,2}, ..., q_{\alpha,U})$. We set $U = 9$ and randomly let 5 elements of $\mathbf{p}_i$ and $\mathbf{q}_\alpha$ to be 1 and the remaining elements to be 0. Each time we randomly select a user i to rate an random selected item α and the rating reads $r_{i\alpha} = \mathbf{p}_i\mathbf{q}_\alpha$. According to the definition, $r_{i\alpha}$ ranges from 1 to 5. In this paper, we let the number of ratings equal to $0.1 \times 500 \times 500$.

Based on the item attribute vectors, one can get the "true" similarity between item α and item β as,

$$s_{\alpha\beta}^{\mathrm{true}} = \frac{\mathbf{q}_\alpha \mathbf{q}_\beta^T}{\sqrt{(\mathbf{q}_\alpha \mathbf{q}_\alpha^T)(\mathbf{q}_\beta \mathbf{q}_\beta^T)}}. \quad (1)$$

One can get their *cosine* similarity $s_{\alpha\beta}^{\cos}$ based on the artificial rating matrix $R = \{r_{ia}\}$. We also compute their MDS similarity $s_{\alpha\beta}^{\mathrm{mds}}$. All the similarities are normalized:

$$\widehat{s}_{\alpha\beta} = \frac{s_{\alpha\beta} - \min(s)}{\max(s) - \min(s)}, \quad (2)$$

where $\max(s)$ and $\min(s)$ are the maximum and minimum of all the similarities, respectively. To compare the *cosine* and *MDS* method, we compute the *mean absolute error* between $\widehat{s}^{\mathrm{true}}$ and $\widehat{s}^{\cos}$ or $\widehat{s}^{\mathrm{mds}}$ for all item pairs. That is,

$$\delta = \frac{2\sum_{\alpha\neq\beta} \mid \widehat{s}_{\alpha\beta}^{\mathrm{true}} - \widehat{s}_{\alpha\beta}^{*} \mid}{N(N-1)}, \quad (3)$$

where $N = 500$ is the number of items. $\widehat{s}_{\alpha\beta}^{*}$ denotes the $\widehat{s}_{\alpha\beta}^{cos}$ or $\widehat{s}_{\alpha\beta}^{mds}$.

The results of δ are presented in Fig. S1. We compare these two methods on the unweight (the left figure of Fig. S1) and the weight (the right figure of Fig. S1) adjacency matrix. For the unweight adjacency matrix, we set $r_{i\alpha} = 1$ if $r_{i\alpha} > 0$ in R. The dimension of Y is increased from 2 to 50. In Fig. S1, there are three main findings: Firstly, the δ of both the MMDS and NMDS decreases with their dimension H. That's to say, it is more accurate to compute the similarities of item pairs by the MMDS and NMDS when using a higher dimension. Secondly, The MMDS is slightly better than the NMDS in computing the item similarity as the δ of MMDS is smaller than that of NMDS. However, their differences tend to be smaller when the dimension increases. Thirdly, both the MMDS and NMDS is significantly more accurate than the cosine method in both unweighted and weighted networks.

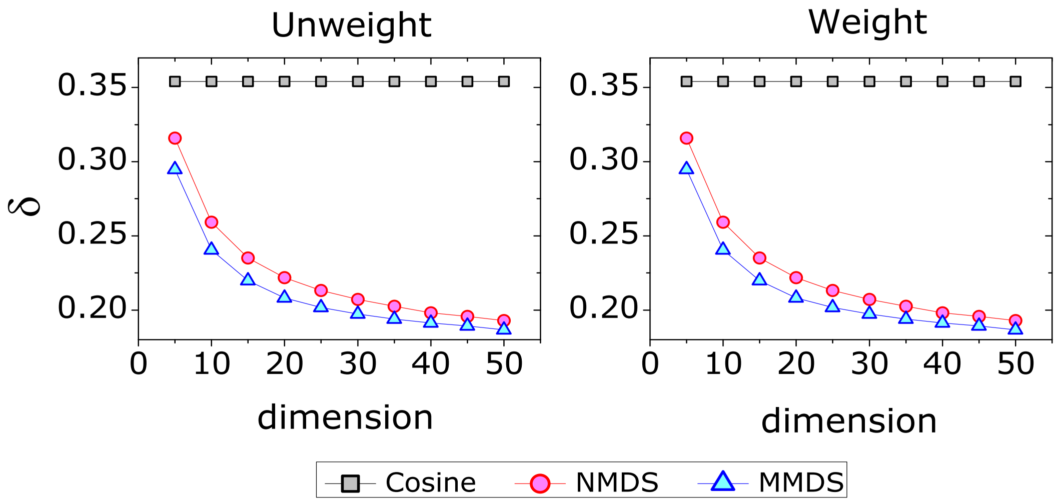

Figure S1. The compare of *cosine* and *MDS* (MMDS and NMDS) method in artificial data. The dimension H of Y is increased from 2 to 50.

Dimension of the MDS method

The relationship between the accuracy (precision and recall) and the dimension of Y computed by the MDS is presented in Fig. S2. One can observe that both the accuracy of ICF-MMDS and ICF-NMDS increases with the dimension when the dimension H is smaller than 100. The slope of the precision and recall curves is

larger when the dimension $H < 30$ and smaller when $H > 30$. Moreover, the ICF-MMDS's accuracy is larger than the ICF-NMDS but their differences gradually become smaller as H increases. The MDS technique aims to place each item in H-dimensional space such that the between-item distances are preserved as well as possible. When H is reduced, a lot of unimportant information containing noise is neglected. Therefore, MDS can be regarded as a noise reduction method. This is shown in the recommendation results in Fig. S2 in which one can see the precision cannot be constantly increases by increasing H (when H is large enough, further enlarging H only includes noisy information).

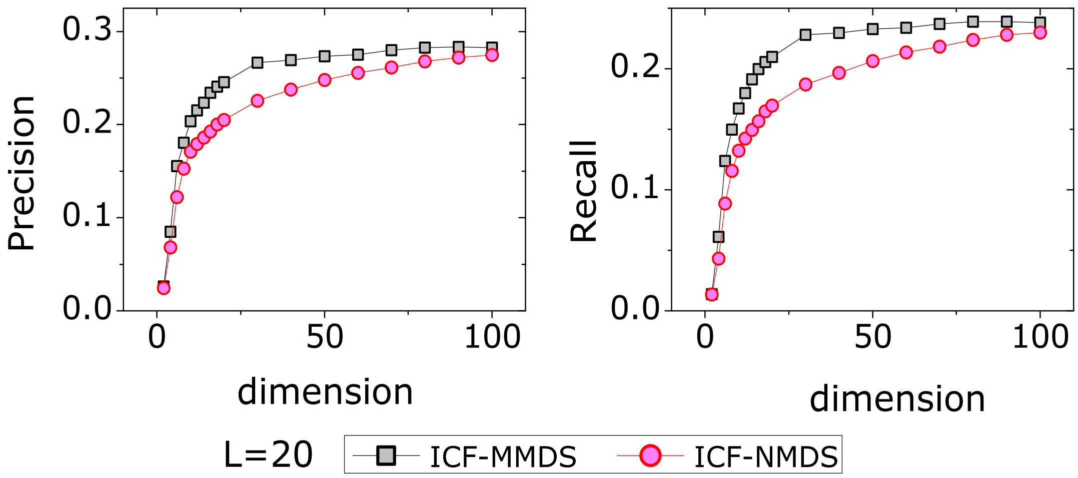

Figure S2. The relationship between the accuracy (precision and recall) and the dimension H of Y computed by the MDS. The recommendation length is 20.

MDS under the user-based CF framework

In this section, we carry out the simulation to compare MDS and cosine similarities under the UCF framework. The results are presented in table S2. Consistent with the manuscript, we here use the precision and recall to measure the recommendation accuracy. UCF-cos and ICF-cos are respectively the user-based CF and item-based CF with nodes' similarity calculated with the cosine method. UCF-MDS and ICF-MDS are respectively the user-based CF and item-based CF with nodes' similarity calculated with the MMDS method. UMCF is short for the modified UCF which is proposed in [2]. From the table, one can see that the user-oriented CF is worse than the item-oriented CF. The accuracy of both UCF and ICF can be improved by the MDS method. The best method here is the ICF-MDS method.

Table S1. The MDS-based method under the UCF framework. The dimensions of ICF-MDS and UCF-MDS are 100 and 200, respectively.

Method	Precision($L = 10$)	Precision($L = 20$)	Recall($L = 10$)	Recall($L = 20$)
UCF-cos	0.2214	0.1767	0.0889	0.1331
MUCF	0.2371	0.1943	0.0971	0.1493
UCF-MDS	0.2993	0.2445	0.1124	0.1683
ICF-cos	0.2929	0.2323	0.1254	0.1853
ICF-MDS	0.3507	0.2844	0.1604	0.2412

Definition of distances in MDS

Apart from the Euclidean distance, we consider here two other definitions of distances:

Euclidean Commute-Time Distance (ECTD). Denote by $m(\alpha,\beta)$ the average number of steps required by a random walker starting from item α to reach item β, the average commute time between α and β is $dist(\alpha,\beta) = m(\alpha,\beta) + m(\beta,\alpha)$, which can be obtained in the terms of pseudoinverse of the Laplacian matrix, $L^+(L = D - B)$, as $dist(\alpha,\beta) = V(l^+_{xx} + l^+_{yy} - 2l^+_{xy})$, where l^+_{xy} is the entry in L^+. B is the adjacent matrix projected by the user-item network with the element $b_{\alpha\beta} = |\Gamma_\alpha \bigcap \Gamma_\beta|$, where Γ_α is the user set who have collected item α. D is a diagonal matrix with $d_{\alpha\alpha} = [D_{\alpha\alpha}] = \sum_{\beta=1}^{N} b_{\alpha\beta}$.

Table S2. MDS based on different distance computations.

Method	Precision($L = 10$)	Precision($L = 20$)	Recall($L = 10$)	Recall($L = 20$)
MMDS-ECTD	0.0476	0.0402	0.0202	0.0329
MMDS-HD	0.2771	0.2236	0.1304	0.1972
MMDS-ED	0.3507	0.2844	0.1604	0.2412

Hamming Distance (HD). $Dist(\alpha,\beta) = c/N$, where c is the number of different users between the user sets of item α and β.

The results are presented in table S3 where one can see that the performance of recommendation method indeed depends significantly on the definition of distance. The best definition for MMDS method is the Euclidean Distance which is the one we used in the manuscript.

Accuracy in Netflix and RYM datasets

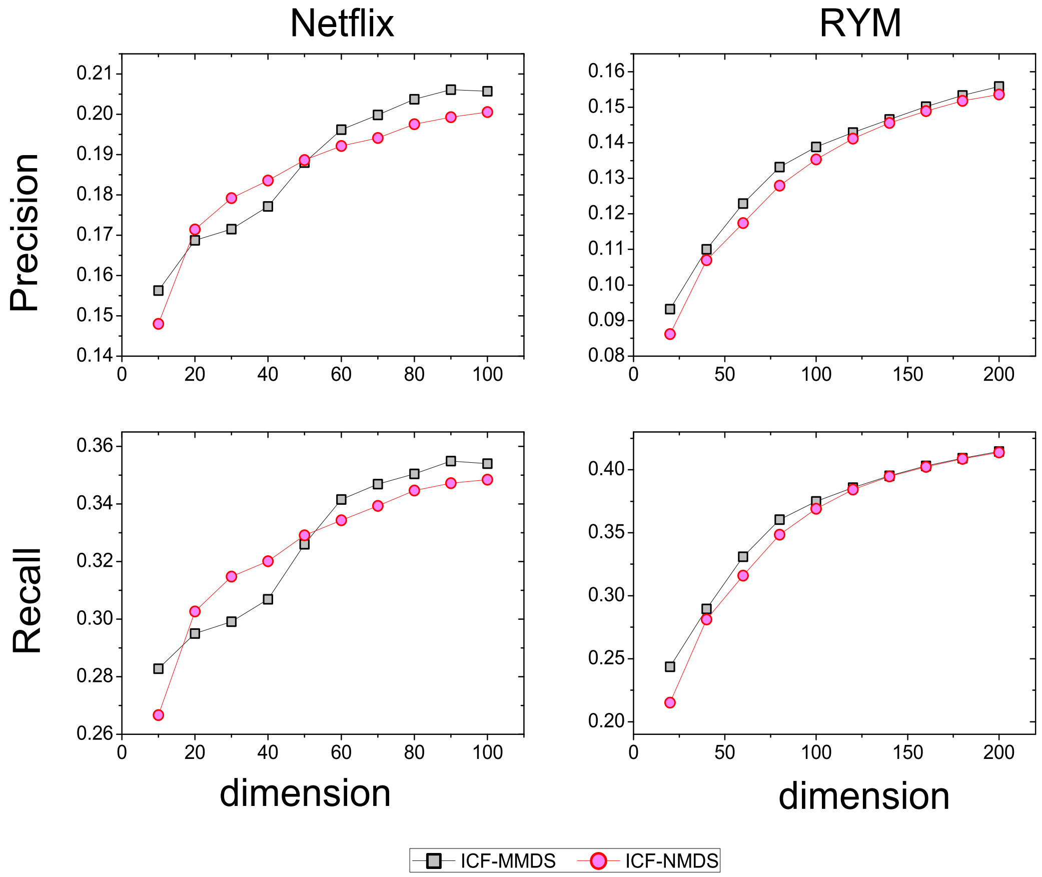

Figure S3. The relationship between the accuracy (precision and recall) and the dimension of Y computed by the MDS on Netflix and RYM dataset. The recommendation length L is 20.

Table S2. The accuracy compare results of different recommendation approaches on Netflix and RYM dataset. The recommendation length is set to 10 and 20. The dimensions of ICF-MMDS and ICF-NMDS are 100 for Netflix and 200 for RYM, respectively. The λ of Hybrid method is 0.2 for Netflix and 0.3 for RYM, respectively.

Netflix dataset				
Method	Precision($L = 10$)	Precision($L = 20$)	Recall($L = 10$)	Recall($L = 20$)
ICF-MMDS	**0.2683**	**0.2057**	**0.2352**	**0.3540**
ICF-NMDS	0.2666	0.2005	0.2355	0.3484
MD	0.2316	0.1820	0.2028	0.3171
HC	0.0003	0.0004	0.0004	0.0009
Hybrid	0.2613	0.2009	0.2356	0.3526
ICF-cosine	0.2417	0.1915	0.2072	0.3270
RYM dataset				
Method	Precision($L = 10$)	Precision($L = 20$)	Recall($L = 10$)	Recall($L = 20$)
ICF-MMDS	**0.2194**	**0.1558**	**0.3177**	**0.4146**
ICF-NMDS	0.2165	0.1536	0.3158	0.4137
MD	0.1567	0.1343	0.2317	0.3730
HC	0.0759	0.0765	0.1255	0.2291
Hybrid	0.2113	0.1530	0.3065	0.4068
ICF-cosine	0.2000	0.1393	0.2988	0.3859

Diversity in Netflix and RYM datasets

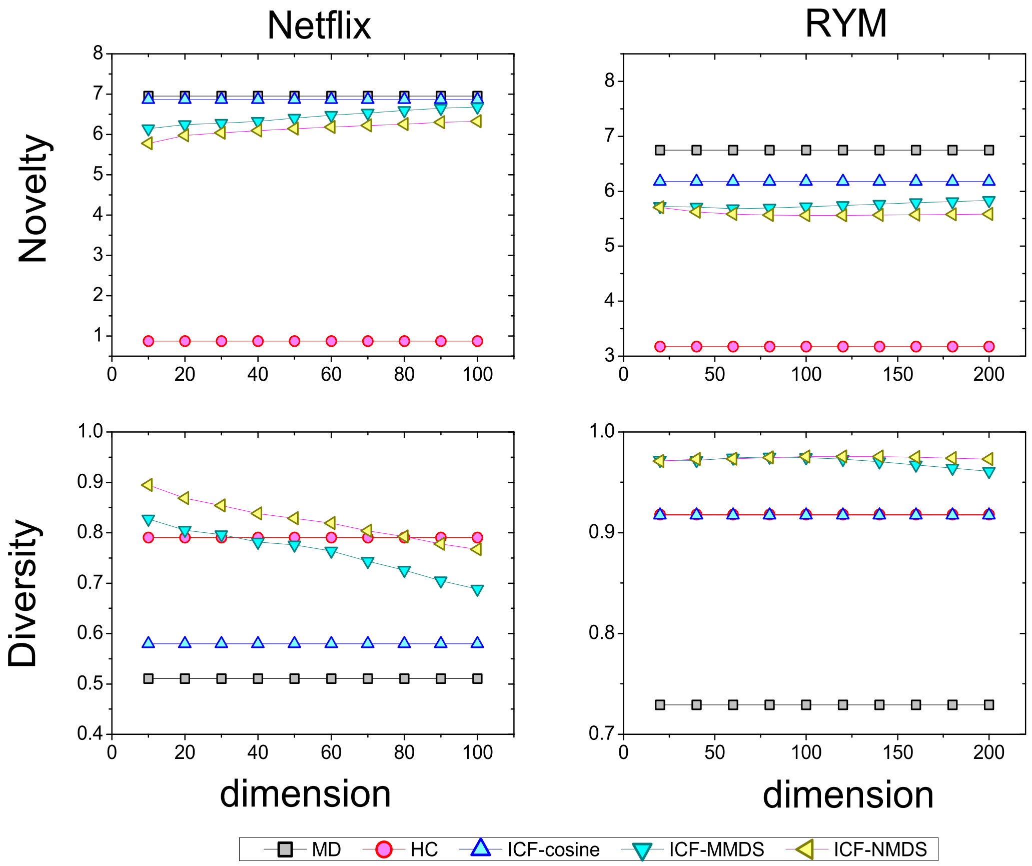

Figure S4. The diversity and novelty results on the Netflix and RYM data. The recommendation length L is 20.

References

[1] Medo M, Zhang YC, Zhou T (2009) Adaptive model for recommendation of news. EPL 88: 38005.

[2] Liu RR, Jia CX, Zhou T, Sun Duo, Wang BH (2009) Personal recommendation via modified collaborative filtering. Physica A 388: 462–468.
